# 76865 Clinical Outcomes of Chronic Myelomonocytic Leukemia in the VA Healthcare System

**DOI:** 10.1017/cts.2021.487

**Published:** 2021-03-30

**Authors:** Rohan Achar, Talha Anwar, Brian Parkin

**Affiliations:** University of Michigan, VA Ann Arbor Health System

## Abstract

ABSTRACT IMPACT: Through a comprehensive analysis of patterns of care and outcomes in Veterans with chronic myelomonocytic leukemia, we will identify Veteran-specific determinants of survival that will allow for more personalized decision-making in this underserved population. OBJECTIVES/GOALS: Little is known about outcomes of Veterans with chronic myelomonocytic leukemia (CMML), a malignancy with high morbidity and mortality. In this study, we will describe patterns of care, identify factors that impact survival, and compare outcomes in this cohort to those of the civilian population. METHODS/STUDY POPULATION: We will conduct a comprehensive retrospective review of approximately 1,000 Veterans with CMML. We will construct a database of demographics, clinical characteristics, disease characteristics, treatment regimens, and outcomes in this cohort. Adult Veterans with a diagnosis of CMML determined by ICD-O-3 coding who were treated through the VA after 1990 and have sufficient electronic health data will be included in this study. Veterans receiving the majority of their care for CMML at non-VA hospitals or clinics will be excluded. Data on veterans will be obtained and validated from VA clinical databases and chart review. Data on the civilian population will be obtained from SEER registries. RESULTS/ANTICIPATED RESULTS: We will first describe the baseline patient characteristics and distribution of disease in this cohort and illustrate the landscape of their CMML care. We will subsequently describe the impact of baseline patient characteristics on pathological features of disease, patterns of care, response to therapy, and survival. We anticipate we will identify several Veteran-specific factors that influence treatment and are prognostic or predictive of survival. After drawing conclusions about the Veteran cohort alone, we will compare baseline characteristics and survival outcomes between the Veteran and civilian populations. We predict we will identify significant differences between these two cohorts. DISCUSSION/SIGNIFICANCE OF FINDINGS: This study will help inform Veteran care by identifying clinical features and patient characteristics that are prognostic or predictive of survival. This will open the door for more accurate risk stratification and personalized treatment that could improve outcomes in this underserved population.

